# Determinants and Frequency of Acute Kidney Injury After Endoscopic Retrograde Cholangiopancreatography

**DOI:** 10.7759/cureus.111623

**Published:** 2026-06-27

**Authors:** Muhammad Minhal Sandhu, Sohaib Ahmad, Muhammad Nibras Mehmood Randhawa, Zailah Mehmood Randhawa, Naimal Mehmood Randhawa, Hafiz Zunair Iqbal, Jahangeer Ahmed, Ammar Noor, Muhammad Irfan Jamil

**Affiliations:** 1 Acute Medicine, Southend University Hospital NHS Foundation Trust, Southend, GBR; 2 Medicine, Pakistan Institute of Medical Sciences, Islamabad, PAK; 3 Medicine, Islamabad Medical & Dental College, Islamabad, PAK; 4 Medicine, Rawalpindi Medical University, Rawalpindi, PAK; 5 Internal Medicine, Akhtar Saeed Medical and Dental College, Lahore, PAK; 6 Gastroenterology, Lahore General Hospital, Lahore, PAK; 7 Internal Medicine, Lahore General Hospital, Lahore, PAK; 8 Nephrology, Lahore General Hospital, Lahore, PAK

**Keywords:** acute kidney injury, endoscopic retrograde cholangiopancreatography (ercp), estimated glomerular filtration rate, post-ercp pancreatitis (pep), risk factors

## Abstract

Background: Endoscopic retrograde cholangiopancreatography (ERCP) is commonly performed for pancreaticobiliary disorders. However, renal dysfunction may occur after the procedure and can influence clinical outcome. This study was planned to estimate the frequency of acute kidney injury (AKI) following ERCP and to assess the clinical and laboratory factors associated with its occurrence.

Methods: This observational analytical study was conducted at the Department of Gastroenterology, Lahore General Hospital, Lahore, from January 2025 to March 2026. A total of 296 patients undergoing ERCP for choledocholithiasis, obstructive jaundice, biliary stricture, or pancreaticobiliary malignancy were enrolled by non-probability consecutive sampling. Patients with end-stage renal disease, pre-existing AKI, prior renal replacement therapy, renal transplant, or unclear repeat ERCP records were excluded.

Results: AKI was observed within 48 hours after ERCP in 52 (17.6%; 95% CI 13.7-22.3%) patients. Most AKI cases were KDIGO Stage 1 (37/52, 71.2%), followed by Stage 2 (9/52, 17.3%) and Stage 3 (6/52, 11.5%). Patients who developed AKI had a higher mean age than those without AKI (61.2 ± 13.8 vs. 52.4 ± 14.9 years, p = 0.001). The Charlson Comorbidity Index (CCI) was also significantly raised in the AKI group (5 (IQR 3-6) vs. 3 (IQR 2-4); p < 0.001). Acute cholangitis was the commonest ERCP indication among patients with AKI as compared with those without AKI (31/52 (59.6%) vs. 58/244 (23.8%), p < 0.001). Baseline renal function was worse among patients who developed AKI, with higher serum creatinine (1.42 ± 0.68 vs. 0.84 ± 0.31 mg/dL) and lower estimated glomerular filtration rate (58.4 ± 28.7 vs. 84.6 ± 24.3 mL/min/1.73 m²), with both comparisons showing statistical significance (p < 0.001). Mean hospital stay was also longer in patients with AKI (9.4 ± 5.1 vs. 6.1 ± 3.6 days, p < 0.001). On multivariable analysis, CCI, acute cholangitis, reduced eGFR, raised bilirubin, low albumin level, and exposure to nephrotoxic drugs or intravenous contrast were identified as independent determinants of post-ERCP AKI.

Conclusion: Post-ERCP AKI is an important renal complication and was mainly associated with greater comorbidity burden, acute cholangitis, reduced baseline renal function, raised bilirubin, low serum albumin, and prior nephrotoxic drug or intravenous contrast exposure. Careful pre-procedural renal assessment and early post-procedural monitoring may help identify high-risk patients and reduce adverse clinical outcomes.

## Introduction

Endoscopic retrograde cholangiopancreatography (ERCP) is an established therapeutic technique for pancreaticobiliary diseases and is frequently performed for choledocholithiasis, obstructive jaundice, biliary stricture, acute cholangitis, and pancreaticobiliary malignancy [1]. Although ERCP has replaced many surgical and purely diagnostic procedures, it remains an invasive intervention with measurable post-procedural risk [2]. Bishay et al. reported that the pooled frequency of post-ERCP pancreatitis was 4.6%. The same study reported bleeding in 1.5%, cholangitis in 2.5%, cholecystitis in 0.8%, perforation in 0.5%, and ERCP-attributable mortality in 0.2% of patients [3]. These figures show that ERCP-related complications are not limited to pancreatic or gastrointestinal events and systemic complications require equal clinical attention.

Acute kidney injury (AKI) after ERCP is an important but relatively under-reported complication [4]. It may occur through several overlapping mechanisms, including baseline renal impairment, biliary sepsis, acute cholangitis, dehydration, raised bilirubin, systemic inflammation, nephrotoxic drug exposure, recent intravenous contrast use, and procedure-related complications [5]. Gadalean et al. reported post-ERCP AKI in 26% of patients, including stage 1 AKI in 19.7% and stage 2 or 3 AKI in 6.3%. Baseline eGFR, Charlson Comorbidity Index (CCI) score, choledocholithiasis, and admission bilirubin level were identified as independent predictors of AKI [6].

The relationship between biliary infection, renal injury, and adverse outcomes is also supported by recent evidence from acute cholangitis literature. Tang et al. reported that AKI occurred in 24% patients with acute cholangitis and was related to more complications, greater need for invasive treatment, longer hospitalization, and increased mortality [7]. Similarly, Lee et al. found AKI in 18.2% of patients hospitalized with acute cholangitis, with a higher risk among older patients and those with hypertension, severe cholangitis, or systemic inflammatory response [8]. In patients with advanced chronic kidney disease (CKD) undergoing ERCP, Baydoun et al. 2026 reported increased odds of bleeding, cholangitis, intensive care admission, post-procedure intubation, and mortality compared with patients having normal renal function [9]. Despite these findings, many ERCP studies still focus mainly on pancreatitis, bleeding, and perforation, while renal outcomes are less frequently assessed. Therefore, the present study was conducted to determine the frequency of AKI after ERCP and to identify its clinical, laboratory, exposure-related, and procedure-related predictors.

## Materials and methods

**Figure 1:**
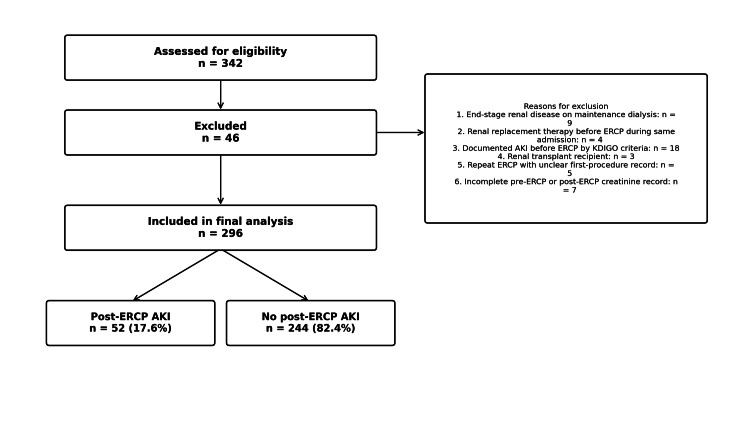
STROBE flow diagram of patient selection AKI: acute kidney injury; ERCP: endoscopic retrograde cholangiopancreatography; KDIGO: Kidney Disease: Improving Global Outcomes

This observational analytical study was executed at the Department of Gastroenterology, Lahore General Hospital, Lahore, Pakistan, from January 2025 to March 2026. The study was conducted after approval from the Ethical Review Committee of Post Graduate Medical Institute/Amer-Ud-Din Medical College/Lahore General Hospital, Lahore (IRB #196). A sample size of 296 was calculated by taking the frequency of acute kidney injury after ERCP as 26% at a 95% confidence level and 5% absolute precision [6]. A non-probability consecutive sampling technique was used to enroll patients. Patient's selection criteria are reported in Table 1 and Figure 1.

**Table 1:** Eligibility criteria of the study population

Inclusion criteria	Exclusion criteria
Patients aged 18 years or older	Patients with end-stage renal disease already receiving dialysis
Patients of either gender	Patients who required renal replacement therapy before ERCP during the same hospital admission
Patients undergoing endoscopic retrograde cholangiopancreatography (ERCP)	Patients with documented acute kidney injury before endoscopic retrograde cholangiopancreatography according to the Kidney Disease: Improving Global Outcomes criteria [10]
ERCP performed for choledocholithiasis, obstructive jaundice, biliary stricture, and pancreaticobiliary malignancy	Renal transplant recipients who underwent repeat endoscopic retrograde cholangiopancreatography, where the first procedure record could not be clearly identified

Data were collected on a structured proforma by the principal investigator or a trained member of the research team. Before ERCP, demographic variables, including age, gender, and body mass index (BMI), were documented. Comorbidity was evaluated using the Charlson Comorbidity Index (CCI) [11]. Clinical status before ERCP was reviewed for the presence of acute cholangitis (diagnosed according to clinical, laboratory, and imaging findings) at presentation. Exposure to nephrotoxic drugs or intravenous contrast before ERCP was also recorded. Baseline laboratory values were recorded from the latest available blood sample taken before ERCP, preferably within 24 hours before the procedure. The recorded laboratory variables included serum urea, serum creatinine, eGFR, total bilirubin, aspartate aminotransferase (AST), alanine aminotransferase (ALT), alkaline phosphatase, white blood cell count (WBC), serum albumin, and C-reactive protein (CRP). All biochemical tests were done in the hospital laboratory as stated by standard operating procedures. Peri-procedural fluid management was carried out according to routine institutional practice. Patients received intravenous isotonic fluids during fasting and around the procedure as clinically indicated.

ERCP was performed by a consultant gastroenterologist under standard institutional protocol. The procedure was carried out after routine pre-procedural assessment, fasting, review of coagulation profile, where clinically required, and optimization of the patient’s hemodynamic and medical status. Sedation or anesthesia was administered according to the patient’s clinical condition. A side-viewing duodenoscope was used for the procedure. Any immediate adverse event observed during or immediately after the procedure was documented. After ERCP, patients were monitored according to routine post-procedure care. The primary outcome was post-ERCP acute kidney injury, defined according to KDIGO criteria as an increase in serum creatinine by ≥0.3 mg/dL within 48 hours after ERCP. AKI severity was classified as KDIGO Stage 1, Stage 2, or Stage 3 [10]. Secondary clinical outcomes included post-ERCP pancreatitis, bleeding, perforation, and hospital stay.

The collected data were processed in IBM SPSS Statistics for Windows, version 26.0 (released 2019, IBM Corp., Armonk, NY). Numerical data were summarized as mean with standard deviation, while categorical data were described as frequency and percentage. Mean differences between groups were assessed by applying the independent samples t-test. Categorical associations were examined using the chi-square test, and Fisher’s exact test was used where cell counts were small. To evaluate factors related to post-ERCP acute kidney injury, univariable logistic regression was first applied. Factors showing significance or having clinical importance were then included in a multivariable binary logistic regression model to determine independent predictors of AKI. The strength of association was presented as crude odds ratio and adjusted odds ratio, along with 95% confidence interval (CI). A p-value of 0.05 or less was taken as statistically significant.

## Results

A total of 296 patients who underwent ERCP were counted in the final analysis. Post-ERCP acute kidney injury was documented in 52 patients (17.6%; 95% CI 13.7-22.3%) within 48 hours after the procedure. The mean age was 54.3 ± 14.7 years, with males constituting 164 patients (55.4%) and females, 132 patients (44.6%). The detailed baseline characteristics are presented in Table 2.

**Table 2:** Baseline demographic and clinical characteristics stratified by the acute kidney injury status (n = 296). Values are presented as mean ± standard deviation (SD), median (interquartile range (IQR)), and number (percentage). P-values were derived using the independent-samples t-test for continuous and the Pearson chi-square test for categorical variables. AKI: acute kidney injury; ERCPL endoscopic retrograde cholangiopancreatography

Variable	Overall (n = 296)	AKI group (n = 52)	No AKI group (n = 244)	p-value
Age (years), mean ± SD	54.3 ± 14.7	61.2 ± 13.8	52.4 ± 14.9	0.001
Sex (female), n (%)	132 (44.6)	20 (38.5)	112 (45.9)	0.327
Body mass index (kg/m²), mean ± SD	25.8 ± 4.2	26.1 ± 4.6	25.7 ± 4.1	0.534
Charlson Comorbidity Index score, median [IQR]	3 [2–5]	5 [3–6]	3 [2–4]	<0.001
Nephrotoxic drug or IV contrast exposure before ERCP, n (%)	78 (26.4)	20 (38.5)	58 (23.8)	0.029
ERCP indication, n (%)				0.856
Choledocholithiasis	158 (53.4)	30 (57.7)	128 (52.5)	
Obstructive jaundice	72 (24.3)	12 (23.1)	60 (24.6)	
Biliary stricture	38 (12.8)	5 (9.6)	33 (13.5)	
Pancreaticobiliary malignancy	28 (9.5)	5 (9.6)	23 (9.4)	
Acute cholangitis at presentation, n (%)	89 (30.1)	31 (59.6)	58 (23.8)	<0.001
Duration of ERCP (hours), mean ± SD	0.68 ± 0.21	0.72 ± 0.24	0.67 ± 0.20	0.148

Baseline serum creatinine was markedly elevated in the AKI group (1.42 ± 0.68) compared with the non-AKI group (0.84 ± 0.31; p < 0.001), while baseline eGFR was correspondingly lower among patients who subsequently developed AKI (58.4 ± 28.7 vs. 84.6 ± 24.3; p < 0.001). The detailed baseline laboratory parameters are presented in Table 3.

**Table 3:** Baseline laboratory parameters of the study population, stratified by the acute kidney injury status (n = 296). Values are presented as mean ± SD. eGFR: estimated glomerular filtration rate; AKI: acute kidney injury

Variable	Normal reference range	Overall (n = 296)	AKI group (n = 52)	No AKI group (n = 244)	p value
Serum urea (mg/dL)	15-45	35.1 ± 18.4	47.8 ± 22.4	32.4 ± 15.8	<0.001
Serum creatinine (mg/dL)	0.6-1.2	0.94 ± 0.50	1.42 ± 0.68	0.84 ± 0.31	<0.001
eGFR (mL/min/1.73 m²)	≥90	79.8 ± 27.9	58.4 ± 28.7	84.6 ± 24.3	<0.001
Total bilirubin (mg/dL)	0.2-1.2	7.0 ± 6.6	10.8 ± 8.4	6.2 ± 6.1	<0.001
Alanine aminotransferase (IU/L)	7-56	143 ± 120	118 ± 104	147 ± 122	0.084
Aspartate aminotransferase (IU/L)	10-40	117 ± 92	128 ± 112	115 ± 88	0.332
Alkaline phosphatase (IU/L)	44-147	308 ± 196	418 ± 234	278 ± 174	<0.001
White blood cell count (×10⁹/L)	4.0-11.0	10.1 ± 4.7	12.8 ± 5.7	9.5 ± 4.3	<0.001
Serum albumin (g/dL)	3.5-5.0	3.1 ± 0.6	2.78 ± 0.62	3.18 ± 0.58	<0.001
C-reactive protein (mg/L)	<5	40.8 ± 56.2	82.4 ± 87.6	31.2 ± 42.8	<0.001

**Figure 2:**
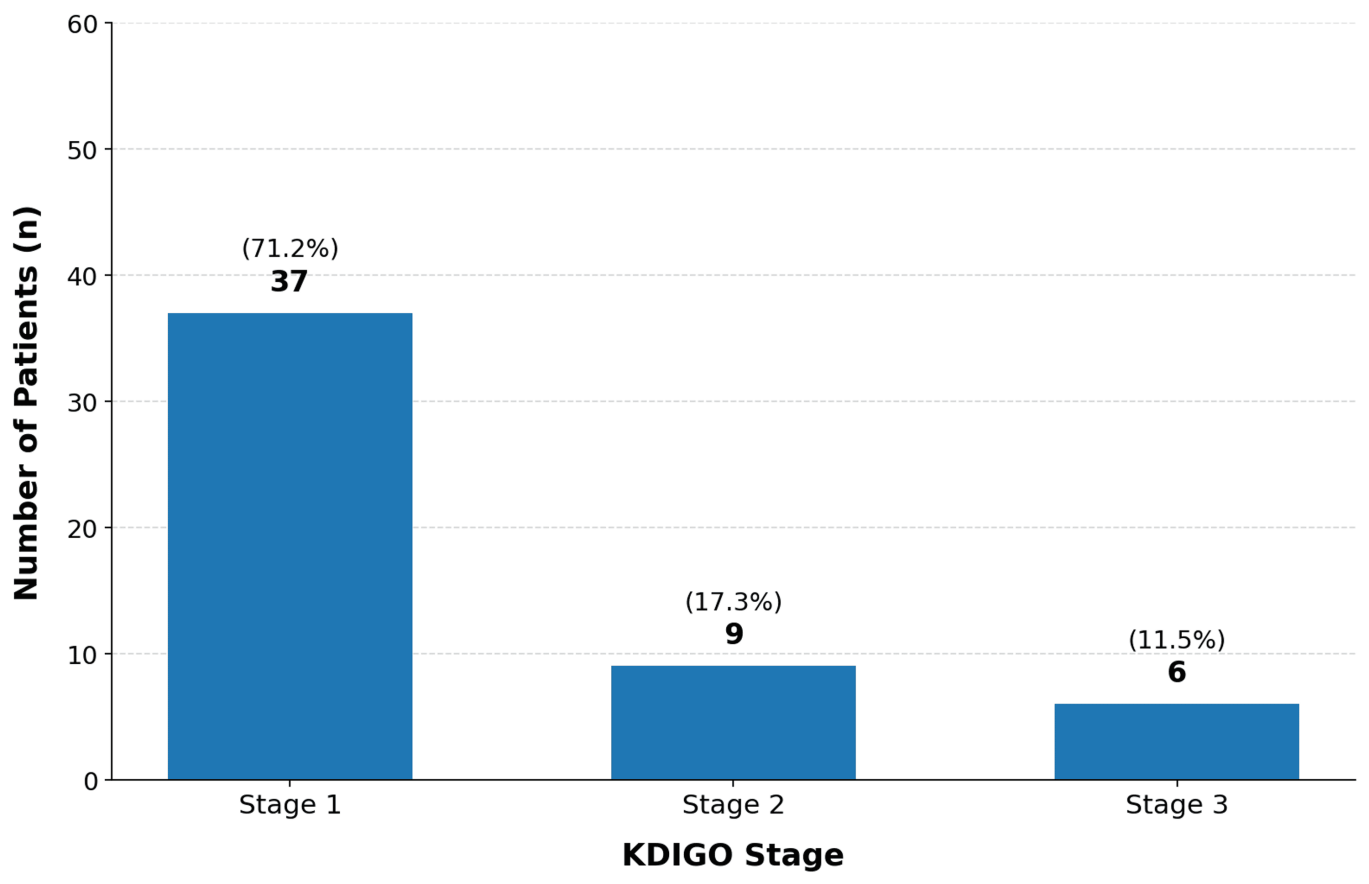
Kidney Disease: Improving Global Outcomes (KDIGO) staging of acute kidney injury.

Of the 52 patients who developed AKI, the majority exhibited KDIGO Stage 1 disease, recorded in 37 patients (71.2%). Stage 2 AKI was identified in nine patients (17.3%), while KDIGO Stage 3 AKI, representing the most severe category of renal impairment, was the least frequent, occurring in six patients (11.5%) (Figure 2). No patient in the study population required renal replacement therapy during the index hospitalization (Figure 2).

Post-ERCP pancreatitis was identified in 18 patients overall (6.1%), with a numerically higher though statistically non-significant frequency in the AKI group (4/52, 7.7%) compared with the non-AKI group (14/244, 5.7%; p = 0.567) (Table 4).

**Table 4:** Post-ERCP complications and length of hospital stay, stratified by the acute kidney injury status ERCP: endoscopic retrograde cholangiopancreatography

Variable	Overall (n = 296)	AKI group (n = 52)	No AKI group (n = 244)	p-value
Post-ERCP pancreatitis, n (%)	18 (6.1)	4 (7.7)	14 (5.7)	0.592
Post-ERCP bleeding, n (%)	8 (2.7)	3 (5.8)	5 (2.0)	0.133
Post-ERCP perforation, n (%)	4 (1.4)	2 (3.8)	2 (0.8)	0.086
Length of hospital stay (days), mean ± SD	6.7 ± 4.1	9.4 ± 5.1	6.1 ± 3.6	<0.001

On the univariate analysis, post-ERCP AKI was associated with several clinical and biochemical variables, as shown in Table 5. On multivariable analysis, independent determinants included higher CCI (aOR 1.26; p = 0.004), acute cholangitis (aOR 3.84; p < 0.001), lower eGFR (aOR 0.96; p < 0.001), higher bilirubin (aOR 1.07; p = 0.001), and lower albumin (Table 5).

**Table 5:** Univariate and multivariable logistic regression analysis of the determinants of post-ERCP acute kidney injury. OR: odds ratio; CI: confidence interval; eGFR: estimated glomerular filtration rate; IV: intravenous; ERCP: endoscopic retrograde cholangiopancreatography. — = variable not entered into multivariable model (univariate p≥0.10) or not significant after adjustment. *Multivariable model included all variables with univariate p < 0.10; forward conditional binary logistic regression was applied. Bold adjusted OR values denote independent statistical significance (p<0.05). Model calibration: Hosmer–Lemeshow test p = 0.62 (adequate fit); Nagelkerke R² = 0.41.

Variable	Univariate analysis		Multivariable analysis*	
	Crude OR (95% CI)	p-value	Adjusted OR (95% CI)	p-value
Age (per year increment)	1.04 (1.01-1.06)	0.001	—	—
Gender (male vs. female)	1.35 (0.76-2.40)	0.344	—	—
Body mass index (per kg/m²)	1.02 (0.94-1.10)	0.534	—	—
Charlson Comorbidity Index score (per unit)	1.34 (1.18-1.52)	<0.001	1.26 (1.08-1.48)	0.004
Acute cholangitis at presentation	5.04 (2.84-8.96)	<0.001	3.84 (2.02-7.32)	<0.001
Choledocholithiasis	1.28 (0.72-2.28)	0.480	—	—
Obstructive jaundice	0.89 (0.46-1.70)	0.812	—	—
Biliary stricture	0.67 (0.27-1.67)	0.462	—	—
Pancreaticobiliary malignancy	1.03 (0.39-2.71)	0.971	—	—
Serum urea (per mg/dL)	1.03 (1.02-1.05)	<0.001	—	—
Serum creatinine (per mg/dL)	3.76 (2.18-6.48)	<0.001	—	—
eGFR (per mL/min/1.73 m²)	0.97 (0.96-0.98)	<0.001	0.96 (0.94-0.98)	<0.001
Total bilirubin (per mg/dL)	1.08 (1.04-1.12)	<0.001	1.07 (1.03-1.11)	0.001
Alanine aminotransferase (per IU/L)	0.998 (0.995-1.001)	0.084	—	—
Aspartate aminotransferase (per IU/L)	1.001 (0.998-1.004)	0.332	—	—
Alkaline phosphatase (per IU/L)	1.003 (1.001-1.004)	0.001	—	—
White blood cell count (per ×10⁹/L)	1.14 (1.07-1.21)	<0.001	—	—
Serum albumin (per g/dL)	0.38 (0.24-0.60)	<0.001	0.46 (0.28-0.77)	0.003
C-reactive protein (per mg/L)	1.008 (1.004-1.012)	<0.001	—	—
Nephrotoxic drug or IV contrast exposure before ERCP	2.10 (1.16-3.80)	0.018	1.98 (1.02-3.84)	0.043
Duration of ERCP (per hour)	1.18 (0.72-1.96)	0.512	—	—

## Discussion

The present study studied the frequency of AKI following ERCP in a hospital-based population and identified its independent clinical and biochemical determinants through multivariable logistic regression analysis. Post-ERCP AKI was documented in 52 (17.6%) of 296 patients. The observed frequency of 17.6% falls within the range reported by published literature, although notable variability exists across study populations and analytical approaches. Gadalean et al. reported a post-ERCP AKI frequency of 26%, using KDIGO serum creatinine criteria applied within 48 hours [6]. The higher frequency in that study may reflect the older study population (median age 69 years vs. 54.3 years in the current study), a higher number of patients presenting with malignant biliary obstruction, and comparatively higher baseline comorbidity burden. By contrast, two large database analyses by Yang et al. and Nessa et al. reported post-ERCP AKI frequency of 11.48% [12,13]. Furthermore, Bangolo et al. and Mehadi et al. reported post-ERCP AKI rates ranging from 12.7% to 31.4%, with the higher rates observed in patients with concurrent atrial fibrillation and its associated comorbidity burden [14].

Acute cholangitis at presentation was an independent predictor of post-ERCP AKI in the present analysis (aOR 3.84; p < 0.001), a finding that is biologically well-grounded. Cholangiovenous reflux, which occurs when biliary ductal pressure exceeds 25 cmH₂O, promotes direct bacteremia and hepatic venous invasion, further amplifying the systemic inflammatory milieu [15]. This mechanism is consistent with the markedly elevated white blood cell count (12.8 ± 5.7 vs. 9.5 ± 4.3 ×10⁹/L) and CRP (82.4 ± 87.6 vs. 31.2 ± 42.8 mg/L; p < 0.001) observed in AKI patients at baseline. The present finding is also corroborated by Gadalean et al., in which acute cholangitis frequency was higher in AKI patients (79.6% vs. 48.1%) and by Yang et al., in which septicemia was the strongest comorbid predictor of post-ERCP AKI (OR 4.2; p < 0.01) [6,12].

Reduced baseline eGFR was retained as an independent predictor of post-ERCP AKI (aOR 0.96; p < 0.001), confirming that pre-existing chronic kidney disease substantially reduces the renal reserve available to withstand procedural and inflammatory insults. Gadalean et al. independently demonstrated reduced eGFR as a multivariable predictor (aOR 0.95; p < 0.001), with a mean eGFR in the AKI group of 52.75 ± 26.04 [6]. The quantitative concordance between the two studies strengthens the external validity of this observation. An elevated baseline serum creatinine, which was higher in AKI patients in the present study (1.42 ± 0.68 vs. 0.84 ± 0.31), was also identified by Adhikari as a significant determinant of subsequent AKI (p = 0.004) [16].

Greater comorbidity, as quantified by the CCI, was independently related to post-ERCP AKI (aOR 1.26 per unit; p = 0.004). This observation parallels the findings of Gadalean et al., in which the nonrenal CCI independently predicted AKI (aOR 1.21; p = 0.034), underscoring that cumulative multimorbidity reduces the physiological reserve necessary to maintain renal homeostasis under procedural and inflammatory stress [6,17]. Yang et al. corroborated this through Elixhauser comorbidity analysis, in which congestive heart failure (OR 2.2), hypertension (OR 1.4), cirrhosis (OR 1.7), and diabetes mellitus (OR 1.1) each independently predicted post-ERCP AKI [12]. Hyperbilirubinemia was recognized as a self-determining predictor of post-ERCP AKI (aOR 1.07 per mg/dL; p = 0.001). Elevated circulating bile acids employ direct cytotoxic effects on renal cells through the disruption of mitochondrial function and induction of oxidative stress. At high concentrations, bile pigments precipitate within tubular lumina, forming bile casts that obstruct nephron flow and provoke tubular [18]. Gadalean et al. reported hyperbilirubinemia as an independent determinant in their study (aOR 1.10-1.12; p < 0.001) [6].

Hypoalbuminemia was associated with a significantly reduced odds of remaining free of AKI (aOR 0.46 per g/dL; p = 0.003), identifying low albumin as a self-determining factor in the present analysis. The mean serum albumin was markedly lower in the AKI group (2.78 ± 0.62 vs. 3.18 ± 0.58 g/dL; p < 0.001). Albumin maintains plasma oncotic pressure, which is fundamental to effective renal perfusion; its depletion results in reduced effective circulating volume and attenuated glomerular filtration [19]. In addition, albumin subserves nephroprotective functions including binding of toxins and nephrotoxic drugs, scavenging of reactive oxygen species, and preservation of proximal tubular integrity [20]. These mechanisms are consistent with published meta-analytic evidence, which has established hypoalbuminemia as a predictor of AKI across a broad range of clinical contexts. In a study, lower albumin was associated with AKI on both univariate and mortality analyses (p < 0.001), further affirming its importance as a biochemical determinant [21].

Pre-procedural exposure to nephrotoxic drugs or intravenous contrast material nearly doubled the odds of post-ERCP AKI (aOR 1.98; p = 0.043). Shinoura et al., who demonstrated that ERCP within 72 hours of contrast-enhanced CT was independently associated with contrast-induced nephrotoxicity (CIN) (OR 3.31; p < 0.001), with an overall CIN rate of 17% versus 5.7% in those not undergoing early ERCP [22]. The implication for clinical practice is that pre-procedural medication reconciliation and the avoidance of nephrotoxin exposure in the peri-procedural window represent modifiable targets for renal risk reduction.

Post-ERCP AKI was linked with a lengthy hospital stay (9.4 ± 5.1 vs. 6.1 ± 3.6 days; p < 0.001), consistent with Gadalean et al., who similarly reported a longer median stay among AKI patients (seven vs. five days; p = 0.002), and with the analyses by Bangolo et al. and Mehadi et al., each of which demonstrated substantially increased length of stay associated with post-ERCP AKI [6,14,23]. ERCP-specific complications, including post-ERCP pancreatitis (7.7% vs. 5.7%; p = 0.567), bleeding (5.8% vs. 2.0%; p = 0.124), and perforation (3.8% vs. 0.8%; p = 0.102), were numerically higher in the AKI group. A study reported comparable non-significant differences in post-ERCP pancreatitis and bleeding between groups, suggesting that procedure-specific complications are not primary drivers of renal impairment following ERCP [24].

The main strength of this study is its focused evaluation of acute kidney injury after ERCP using clinically relevant demographic, biochemical, procedural, and post-procedural variables. Use of KDIGO criteria strengthens outcome assessment, while analysis of determinants such as baseline renal function, cholangitis, bilirubin, albumin, comorbidity burden, and nephrotoxic exposure provides practical clinical value. Pre-ERCP and post-ERCP sepsis were not analyzed as separate variables. However, acute cholangitis at presentation was recorded and analyzed as a clinically relevant infection-related variable. However, some limitations should be considered. This was a single-center study with non-probability consecutive sampling; therefore, the findings may not be generalizable to all ERCP settings. AKI was defined mainly by serum creatinine, as urine output documentation was not consistently available. Long-term renal recovery was not assessed. Data on exact nephrotoxic or contrast exposure, peri-procedural intravenous fluids, rectal indomethacin, pancreatic duct stenting, and sepsis severity were limited.

## Conclusions

Post-ERCP AKI occurred in approximately one in six patients undergoing this procedure, with the majority of cases representing mild, non-dialysis-requiring renal impairment. Six independent determinants were established: acute cholangitis at presentation, pre-procedural nephrotoxic or contrast exposure, greater comorbidity burden, hyperbilirubinemia, reduced baseline eGFR, and hypoalbuminemia. These determinants are largely patient-related rather than procedure-specific, underscoring the importance of pre-procedural renal risk stratification in patients scheduled for ERCP. Prospective multi-center studies with standardized AKI surveillance and longer follow-up are necessary to define the long-term renal trajectory of patients sustaining post-ERCP AKI.
